# Letter in Relation to the Case Narrated by Dr. Ruschenberger, in the Second Number of This Journal

**Published:** 1842-02-05

**Authors:** B. Ticknor

**Affiliations:** U. S. Naval Hospital, Brooklyn


### *Letter in relation to the case narrated by Dr.* Ruschenberger, *in the second number of this journal.*

To the Editors of the Medical Examiner.

Gentlemen,—In the second number of your periodical, which I received on the 22d inst., is the report of a case of "Deformed Leg from unsuccessfully treated Fracture," on which I wish to offer a few remarks; for I am the surgeon by whom it was the misfortune of Lieut. M—— to be treated. The report of this case leads to the conclusion, that there had been mismanagement on the part of the surgeon; but whether there was or not, yourself and your readers will be better able to judge after being informed of all the particulars of the case; and you will also be able to judge how far the term "unsuccessfully treated" is applicable to it. Every surgeon who has had many fractures to treat, will readily assent to this truth, that there are cases of fracture of the leg which do not deserve to be denominated unsuccessfully treated, though there may be a shortening of half an inch—which is the only deformity that existed in the case of Lieut. M——. With the best treatment that fractures can receive on shore, it happens, not unfrequently, that in bad fractures of the leg there remains as much deformity, at least, as in the case of Lieut. M——.

This was a very bad fracture; oblique, and not transverse, as stated in the report. The soft parts were much lacerated, though the bones did not quite protrude through the skin; and there was a very near approach to gangrene. The sea was rough at the time, as is truly stated in the report, and continued so most of the time, till the ship reached Mahon, on the 4th of January. Those who have treated fractures at sea know the difficulty, even in the most favourable circumstances, in preventing motion at the fracture. In the case of Lieut. M—— there was a nervous susceptibility, and a consequent intolerance of pain, which rendered the management of it much more difficult than it would otherwise have been. No extension could be borne; and the apparatus had to be very frequently readjusted, in order to relieve him from pain. If it be said that a better apparatus might have been employed, I reply, that none could have been used which would wholly have relieved the patient from pain, and which would not consequently have required frequent alteration.

On arriving at Mahon, it become absolutely necessary to take Lieut. M—— on shore, as he could not endure confinement on the orlop-deck any longer. Every precaution was used that I could devise, to have the removal effected without injury to the patient; but it was attended with pain, and the progress of cure was no doubt retarded by it. While on shore efforts were made to produce extension, but they could not be borne. I told Lieut. M——, from the beginning, that there would be some shortening of the limb; and the result was certainly not worse than any one might expect in such a case as his was, taking every thing into consideration. He was never urged by me to use the limb, until I supposed the union was sufficiently firm to warrant it.

Such, gentlemen, are some of the most essential particulars which have been omitted in the report of Lieut. M.'s case, but which ought to be known, in order that it may be judged of correctly. I therefore hope that this communication may be inserted in your Examiner.

I am, respectfully, gentlemen,
Your obed. serv't,
B. Ticknor.

*U. S. Naval Hospital, Brooklyn,* Jan. 25th, 1842.

